# Device Modeling of Efficient PBDB-T:PZT-Based All-Polymer Solar Cell: Role of Band Alignment

**DOI:** 10.3390/polym15040869

**Published:** 2023-02-09

**Authors:** Marwa S. Salem, Ahmed Shaker, Mostafa Mohamed Salah

**Affiliations:** 1 Department of Computer Engineering, College of Computer Science and Engineering, University of Ha’il, Ha’il 55211, Saudi Arabia; 2 Faculty of Engineering, Ain Shams University, Cairo 11535, Egypt; 3 Electrical Engineering Department, Future University in Egypt, Cairo 11835, Egypt

**Keywords:** all-polymer, double HTL, kink effect, PCE, polymerized small molecules

## Abstract

In this study, we present some design suggestions for all-polymer solar cells by utilizing device simulation. The polymer solar cell under investigation is formed by a photoactive film of a blend comprising PBDB-T as a polymer donor and PZT as a polymerized small molecule acceptor. The initial cell is based on a fabricated cell whose structure is ITO/PEDOT:PSS/PBDB-T:PZT/PFN-Br/Ag, which has a power conversion efficiency (PCE) of about 14.9%. A calibration procedure is then performed by comparing the simulation results with experimental data to confirm the simulation models, and the material parameters, implemented in the SCAPS (Solar Cell Capacitance Simulator) simulator. To boost the open circuit voltage, we investigate a group of hole transport layer (HTL) materials. An HTL of CuI or P3HT, that may replace the PEDOT:PSS, results in a PCE of higher than 20%. However, this enhanced efficiency results in a minor S-shape curve in the current density-voltage (J-V) characteristic. So, to suppress the possibility of the appearance of an S-curve, we propose a double HTL structure, for which the simulation shows a higher PCE with a suppressed kink phenomenon due to the proper band alignment. Moreover, the designed cell is investigated when subjected to a low light intensity, and the cell shows a good performance, signifying the cell’s suitability for indoor applications. The results of this simulation study can add to the potential development of highly efficient all-polymer solar cells.

## 1. Introduction

Photovoltaic (PV) devices offer a sustainable solution to deal with the issues of growing energy needs. Several kinds of solar cell technologies have been introduced in the literature. The emphasis is on achieving high power conversion efficiencies (PCEs) along with low-cost solar cells (SCs) [1,2,3]. In this context, research on low-cost silicon-based structures has been published [4,5,6,7,8]; however, the reported PCEs are still low. Thus, new thin-film technologies have emerged, that fulfill both higher efficiencies and lower costs. Currently, the third generation of SCs, including dye-sensitized, perovskite, and bulk-heterojunction SCs, is the most broadly explored and rapidly advanced. In this regard, polymer SCs have enticed substantial interest in recent years, thanks to their lower processing costs, in addition to a manageable process flexibility and being lightweight [9]. Besides, the thicknesses of the films utilized in polymer SCs are reduced because of their high material absorption coefficient [10].

The photoactive film of the polymer SCs is comprised of a p-type conjugated polymer, which acts as a donor, and an n-type organic material, which may be a conjugated polymer acceptor or a small molecule acceptor. Notably, all-polymer SCs consist of both polymer donors and polymer acceptors. The PCEs of polymer SCs have now approached about 18% [11], driven by the persistent development in small molecule acceptors [12,13,14], while all-polymer SC performance still lags behind them. The current PCEs of all-polymer SCs are still lower than those of the small molecule acceptor-based organic solar cells, mainly due to the limited number of polymer acceptors that have a promising performance available for research. This kind of solar cell offers unique characteristics, including exceptional morphological stability and mechanical durability [15,16]. Many efforts have been conducted to provide key design procedures for the advancement of all-polymer SCs.

High-performance all-polymer SCs were obtained when blending PBDB-T polymer donors with Y5 derivatives [17] (the materials’ abbreviations are listed in the supplementary data). However, Y5 derivatives like PYT are restricted by their relatively high optical bandgap values above 1.43 eV, resulting in a limited short-circuit current [18]. On the other hand, Ying et al. developed a polymer acceptor PYF-T which demonstrated a lower optical bandgap value of 1.41 eV, resulting in higher values of short-circuit current density (*J_SC_*) and PCE [19]. Recently, the strategy of polymerizing small molecule acceptors has been introduced, resulting in a drastic rise in the efficiencies of all-polymer SCs [20,21,22,23]. Based on this technique, numerous narrow bandgap n-type doped polymers have been introduced, and PCEs higher than 13% have been reported [20,21,22,23]. More recently, a polymerizing small molecule acceptor based on benzotriazole-core fused, denoted as PZT-C1, was synthesized, and paired with a polymer donor PBDB-T. The resulting all-PSC achieved a record PCE of 14.9% [24].

Apart from this, it has been shown that the hole transport layer (HTL) plays a role in controlling thin film solar cell structures, by engineering the band alignment at the junction interface. Numerous studies on different HTLs for organic solar cells have been performed [25,26,27,28]. PEDOT:PSS is recognized as one of the best polymer materials utilized as an HTL, and especially for inverted SCs thanks to its high conductivity, visible transparency, and low cost [29]. Although PEDOT:PSS provides a well-coordinated work function with ITO, it may not be fully suited to be a high-efficiency partner with the polymer donor because of the mismatched work function between its highest occupied molecular orbital (HOMO) and the corresponding HOMO of the polymer donor. Based on the latter, holes may not be removed effectively from the polymer layer toward the ITO contact, resulting in higher recombination losses. Thus, various alternative HTLs have to be investigated for the proper match with the selected polymer donor. Choosing and designing the most appropriate HTL candidate for the target polymer donor is essential to achieve the maximum possible efficiency. 

The optimization of all-polymer solar cells by employing trial-and-error experimental studies is remarkably expensive and may be unsuccessful. Thus, simulation work is required, as a more efficient technique either to optimize the cell performance and/or to understand the physics beyond the trend of cell parameters when subjected to various conditions. A lot of simulation studies regarding polymer-based solar cells have been published. An analysis demonstrated that a single-junction polymer SC with a P3HT:PCBM photoactive layer can achieve a PCE of 2.9% [30]. A simulation of a cell whose blend is PTB7-Th:PC_71_BM was performed to optimize the thickness of the active layer, revealing an efficiency of 8.15% for a device thickness of 270 nm [31]. Incorporating a non-fullerene ITIC acceptor with a PBDB-T donor has been investigated and the optimization of the cell gave an efficiency of 14.25% [32]. The system ITO/PEDOT:PSS/PT7B:PC70BM/PFN-Br/Ag has been simulated and, upon optimization, a maximum PCE of 8% has been reached [33]. In [34], the PTB7:PC_70_BM blend has been explored with different electron transport layer (ETL) materials, and it was found that Zn(O,S) is the most suitable partner, that achieved a PCE of 17.15%. Some simulation studies inspected the replacement of the conventional PEDOT:PSS with other promising candidates [35,36,37]. Regarding these simulation studies, and many others, all-polymer SCs based on polymerized acceptors have not been investigated by simulation until now, to the best of our knowledge.

Based on the previous discussion, this simulation study focuses on the design of polymer-based SCs, to enhance their performance and pave the way for possible indoor applications, along with other conventional applications. The simulation model implemented is firstly validated against a previously fabricated solar cell, which is based on PBDB-T:PZT and has the configuration ITO/PEDOT:PSS/PBDB-T:PZT/PFN/Ag [24]. While the short-circuit current is relatively high, the initial cell suffers from its relatively low open circuit voltage. So, to improve the open-circuit voltage (*V_OC_*), the replacement of the conventional HTL material with other inorganic and organic candidates has been investigated. Although some HTL materials can achieve higher efficiencies, S-shape behavior in the J-V characteristics can occur due to the misalignment between the valence band edge and ITO contact. So, a double HTL structure has been proposed, in which the PEDOT serves as the HTL near the ITO contact, to accomplish a proper band alignment. In addition, an extra HTL beside the PEDOT is added, to attain a suitable valence band offset for the hole flow. In this work, all analyses have been carried out by SCAPS (Solar Cell Capacitance Simulator) numerical simulation under AM1.5G irradiance [38]. This simulation work introduces a potential route for boosting the all-polymer solar cell efficiency, and therefore it can provide design guidelines for future experimental attempts.

## 2. Simulation Approach and Solar Cell Structure

### 2.1. SCAPS Numerical Approach

In the present simulation study, the SCAPS 3.3.10 simulator is utilized to design and assess the polymer solar cell. SCAPS is a one-dimensional solar cell simulator developed at the department of Electronics and Information Systems (ELIS) of the University of Gent, Belgium. The SCAPS software has been extensively utilized in the modeling and simulation of thin-film devices, including organic and polymer solar cells [32,33,34,39]. The simulator solves Poisson’s equation, coupled simultaneously with electron and hole continuity equations. Then, by applying the drift-diffusion transport model, the electron and hole current densities are evaluated. A flowchart of the simulation techniques, as well as the inputs and main equations, is represented in Figure S1 in the Supplementary Materials. It should be pointed out here that the recombination mechanisms taken into consideration are Shockley–Read–Hall (SRH), direct band-to-band, and Auger recombinations. Besides, defect properties like energy levels and trap densities can be defined in the bulk of the material or at the interface. When defining a defect in the bulk, one identifies bulk minority carrier lifetimes ($\tau_{n}$ and $\tau_{p}$), while recombination velocities, $S_{n}$ and $S_{p}$, characterize the interface defects [40]. The main input and output parameters from SCAPS are listed in Table S1, along with their definitions.

### 2.2. Solar Cell Configuration and Simulation Parameters

**Figure 1:**
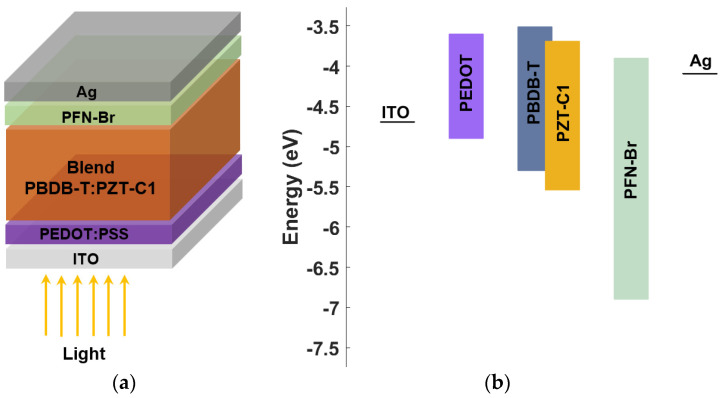
Polymer-based solar cell structure: (**a**) basic layers ITO/PEDOT/PBDB-T:PZT/PFN/Ag, (**b**) energy band profile before contact, showing LUMO and HOMO energy levels in eV.

The device construction of the polymer-based solar cell is demonstrated in Figure 1a. The energy band alignment profile before contact, showing the lowest unoccupied molecular orbital (LUMO) and HOMO (highest occupied molecular orbital) levels relative to the vacuum level of the various thin films, are also displayed in Figure 1b. The solar cell includes the following thin layers. The ITO front contact, which has a work function of about 4.7 eV, PEDOT:PSS as an HTL, and PFN-Br as an ETL. The photoactive layer is a blend of PBDB-T:PZT, where PBDB-T is a donor while PZT-C1 is an acceptor. Finally, the Ag back contact has a work function of about 4.1 eV. All material parameters are indicated in Table 1. The boundary conditions of both contacts are taken to fulfill thermionic emission, with specified electron and hole surface recombination velocities as provided in Table 2 [41]. Electron or hole thermal velocities are considered to be constants for all layers (where *v_th_* = 10^7^ cm/s). The technological parameters of the polymer blend are as follows. The layer thickness is taken to be about 100 nm, as reported experimentally [24]. The carrier mobilities were deduced from the space-charge-limit current (SCLS) [24]. Furthermore, the conduction and valence DOS of all materials were estimated according to reported values concerning organic and polymer solar cells [33,42].

**Figure 2:**
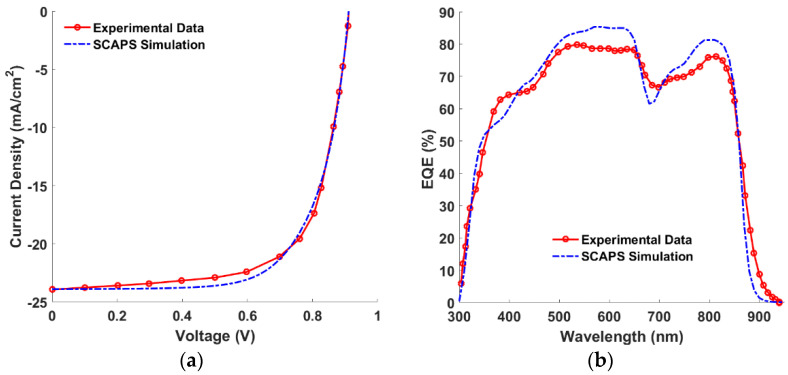
Calibration of calculated vs experimental data (**a**) *J*-V characteristics and (**b**) *EQE* spectra.

**Figure 3:**
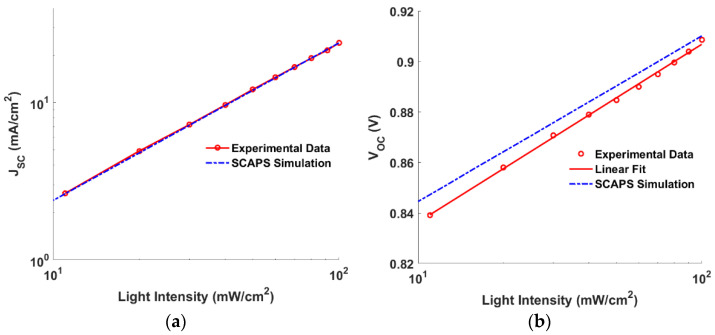
Variation of (**a**) *J*_sc_ and (**b**) *V*_OC_ on light intensity for both experimental and calculated data.

Now, to check the reliability of the physical models and material parameters used in the simulation, the preliminary physical and technological parameters of the distinct thin films are employed to obtain the PV performance parameters. The illuminated current density-voltage (*J-V*) and EQE curves are presented in Figure 2a,b for both the experimental and calculated data, respectively. The PV factors are listed in Table 3, which shows a good match between the calibrated results and those taken from the measurements. Moreover, the dependency of the short-circuit current and open circuit voltage on light intensity is demonstrated in Figure 3a,b, respectively. The behavior of the *J_SC_* for both simulation and measurements is identical, because it is well known that *J_SC_* can be formulated as a function of light intensity (*I*) as given in the following equation [43],

(1) $$J_{SC}=I^{\alpha }+\mathrm{constant},$$

The values of the constant *α* were computed as 0.993 and 0.998 for measured and simulated SCs, respectively. Notably, the closer the *α* value is to unity, the lower the probability of bimolecular recombination [43]. Additionally, regarding the dependence of *V_OC_* on *I*, it can be formulated as [44],

(2) $$V_{OC}=\varepsilon V_{T}ln\left(I\right)+constant,$$

where *V_T_* is the thermal voltage and *ε* is the ideality factor [45]. In the case of the open circuit, where no current flows, all photogenerated charge carriers recombine in the absorber layer; so, the carrier recombination process inside the bulk can be defined [45]. It was found that the cell shows added SRH recombination when *ε* is more than one [46]. The values of *ε* were determined as 1.16 and 1.10 for the measured and simulated SCs, respectively. The previous simulation runs, and their good agreement with the measurements, signify confirmation of the used physical models and material parameters involved in the SCAPS simulation.

## 3. Results and Discussions

To achieve high-performance polymer-based SCs, the materials employed in the design of HTLs need to show some distinct properties. First, a high work function that matches the HOMO level of the polymer donor and the ITO energy level. Second, transparency is required, for the inverted cell to permit higher light absorption in the active layer. Third, high hole mobility is essential to facilitate the hole transport, thereby lowering the charge accumulation and recombination. Finally, a large band gap is important to block electron carriers. Although the conventional PEDOT:PSS fulfills most of the previous criteria, it fails, to a considerable extent, in blocking electron transport, which results in a lower *V_OC_*, as indicated by the previous results. Further, the presented SC is a p-i-n heterostructure in which the heterojunction interface remarkably performs a crucial role, as it controls the recombination, current transport, and the electric field at both sides of the heterojunction. Thus, more investigations about suitable HTL material candidates should be performed.

For the analysis of the HTL, one needs to define the interface barrier between the HTL and the absorber. This can be done by defining the valence band offset (VBO) which is defined as the energy difference between the HOMO level of the HTL and that of the absorber [47]. It was found theoretically that the optimum position of the valence band of the HTL was in the range of −0.1 to 0.2 eV less than the absorber valence band edge [48]. This study was carried out for a perovskite solar cell. We believe that the results of this “optimum” choice do not provide a unique feature for other thin-film SCs. The simulation study was done for a very high interface trap density that may lead to the impedance of some other effects, due to the dominance of the interface recombination. So, we need to provide an independent analysis to design a suitable VBO for our specific SC. 

**Figure 4:**
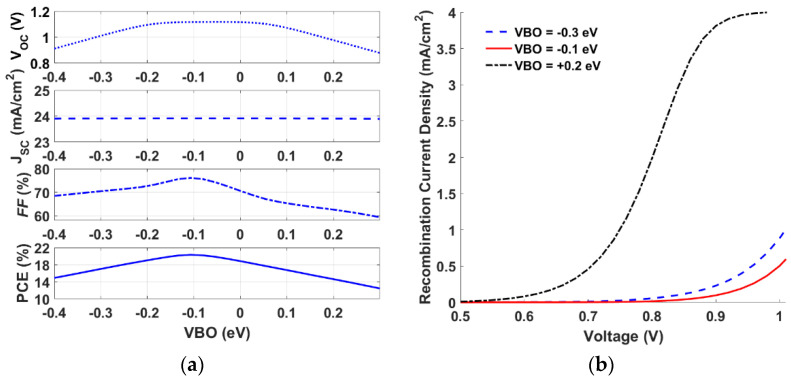
Impact of VBO: (**a**) Variation of PV performance factors versus VBO, and (**b**) Interface recombination current for different values of VBO.

Herein, we start with a theoretical study in which we vary the bandgap of PEDOT HTL while keeping all other parameters as listed in Table 1. This variation results in a valence band offset change from −0.4 to 0.3 eV. By applying this variation, we can also achieve suitable electron blocking along with a proper VBO. This study gives a figure of merit of the trend of PV cell parameters concerning the variation of the VBO. As indicated in Figure 4a, the PV performance parameters vary with the change in the VBO. An optimum efficiency occurs at VBO = −0.1 eV. The PCE is improved because of the fill factor (FF) and *V_OC_* enhancements, while the *J_SC_* does not change for the various values of the VBO. To assess this trend, the interface recombination current is plotted for the three cases of the VBO, as displayed in Figure 4b. As can be inferred from the figure, the interface recombination is higher for VBO = −0.3 and +0.2 eV, whereas the recombination is lower for the optimum value of the VBO (−0.1 eV).

### 3.1. Impact of HTL Materials

**Figure 5:**
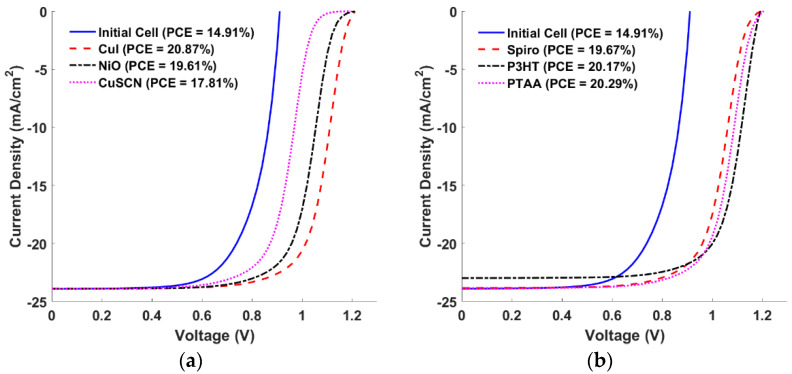
J-V curves for different materials of a single HTL. (**a**) Inorganic and (**b**) Organic.

Generally, the HTL materials applied in polymer SCs can be categorized into inorganic and organic materials. Table 4 presents the basic physical parameters of some inorganic and organic HTL materials. The chosen materials were selected to scan various values of VBO for both inorganic and organic materials. Inorganic HTMs like copper-based CuSCN, CuI, and nickel-based NiO show promising features in organic-based SCs, thanks to their good band alignments and high conductivities, along with other characteristics [49,50]. Further, organic P3HT, PTAA, and Sprio-OMeTAD are also potential candidates that are widely used in organic SCs [50,51,52]. Of the selected materials for the designed all-polymer cell, CuI and P3HT are the most suitable HTMs according to the predictions of the simulation carried out here, as they give a VBO of −0.1 eV with the absorber. However, the majority barrier height between the HTL and the front ITO contact also plays a crucial role and must be taken into consideration. For this reason, the values of the majority (here hole) barrier height (*φ_B_*) are presented in Table 4 for the various HTL materials. It should be pointed out that there are various techniques that could be used for the deposition of HTL materials such as physical vapor deposition, thermal evaporation, e-beam evaporation, sputtering, and molecular beam epitaxy [53]. The different HTL materials, and the corresponding J-V characteristics under illumination, are illustrated in Figure 5. The curves of the inorganic HTLs are represented in Figure 5a, while those of their organic counterparts are shown in Figure 5b. The PV parameters of all studied HTLs are summarized in Table S2 in the Supplementary Materials. The results reveal that CuI and P3HT are the most suitable HTL choices that have minor S-shape curve behavior. Other materials show S-shape curves, especially inorganic materials. Although the P3HT-based cell gives a lower *J_SC_*, its efficiency is higher than that of some other materials. The reduced *J_SC_* is attributed to the low energy gap of P3HT, which is responsible for the absorption of photons before reaching the absorber, thereby lowering the quantum efficiency and thus *J_SC_*. It should be pointed out here that the S-shape occurs due to the band misalignment of the HTL with the ITO contact, which is explained based on the value of *φ_B_*. This misalignment results in an electric field which causes the blocking of hole transport to the contact and triggers more kink behavior. Regarding the inorganic HTL materials, CuSCN has the highest *φ_B_*, which in turn reflects on the conservable S-shape appearance. The lower S-shape behavior corresponds to CuI, which has the lowest *φ_B_* as indicated in Table 4. Moreover, concerning the organic HTL materials, it is noted that P3HT has the lowest S-shape trend, although its *φ_B_* is not the lowest among the other partners. This is due to the hole mobility having the highest value compared to the other organic HTL materials. Finally, the hole transport from the HTL to the front contact not only depends on the barrier height but also on the hole mobility. The overall impact is that the P3HT case has the lowest kink effect.

### 3.2. Proposed Double HTL Structure Design

To fully design the solar cell under investigation, it is preferable to alleviate the possibility of the occurrence of the S-shape curve that is produced due to the misalignment of the HTL material with the work function of the front ITO contact. Also, the VBO should be engineered to achieve the maximum possible PCE. To provide a solution for the kink issue, several attempts have been researched in the literature. One of them is to increase the work function of the ITO by spin-coating cesium-fluoride on the top of the ITO; using this method, an increase in the ITO work function occurs, up to 5.75 eV depending on the concentration of the cesium-fluoride [54]. The other solution is to construct a double HTL layer that is composed of two HTL materials. The HTL adjacent to the ITO should provide an alignment by engineering its HOMO, while the HTL adjacent to the absorber achieves an alignment by the proper choice of the VBO. In this regard, CuI/PEDOT double HTL has been employed experimentally in perovskite solar cells, resulting in a considerable improvement, with a record PCE of 15.75%, in comparison to the reference cell (with PEDOT as an HTL), whose PCE was just 12.5% [55]. Recently, an experimental study on a perovskite solar cell revealed that by the combination of MoS_2_ and PTAA to form a double hole transport layer, the PCE was enhanced to 18.47% in addition to having an improved stability, while the reference cell with PTAA alone achieved a PCE of only 14.48% [56]. The measured improvement was ascribed to the decreased interface resistance and improved hole extraction capability as revealed by electrical impedance and fluorescence spectroscopy measurements [56].

**Figure 6:**
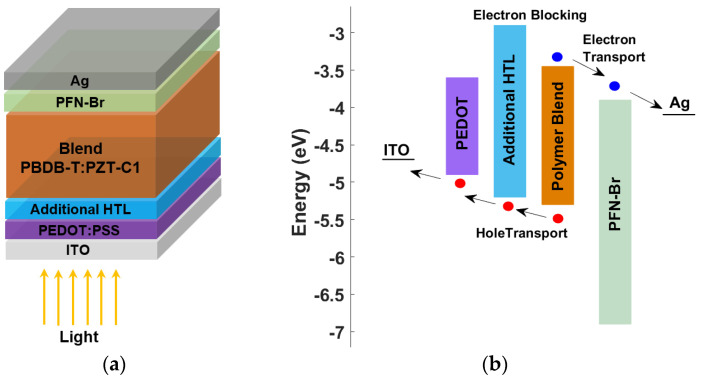
Proposed all-polymer-based solar cell structure: (**a**) basic layers, (**b**) energy band profile before contact.

In this subsection, the incorporation of a double HTL layer that consists of PEDOT as an adjacent layer to the ITO is investigated for various neighboring inorganic and organic materials. The proposed structure is exhibited in Figure 6a, showing the main PEDOT HTL and the additional HTL sandwiched between the PEDOT and the absorber. Figure 6b displays the expected energy level diagram of the double HTL structure, in which it is indicated that holes are transferred via the extra HTL and PEDOT double layer. As can be noticed from the energy profile, the energy difference between the HOMO level of the addition HTL and that of the absorber can be engineered to be low enough to facilitate hole injection. Furthermore, the high difference between the LUMO level of the added HTL and the LUMO level of the blend absorber creates an efficient blocking of electrons, which is translated into reduced charge recombination. Moreover, the difference between the ITO work function and the HOMO level of the PEDOT is well-designed, as proved by the absence of an S-curve behavior when using only PEDOT as an HTL. Thus, the double HTL design concurrently can provide both a higher efficiency and a suppression of the kink effect.

**Figure 7:**
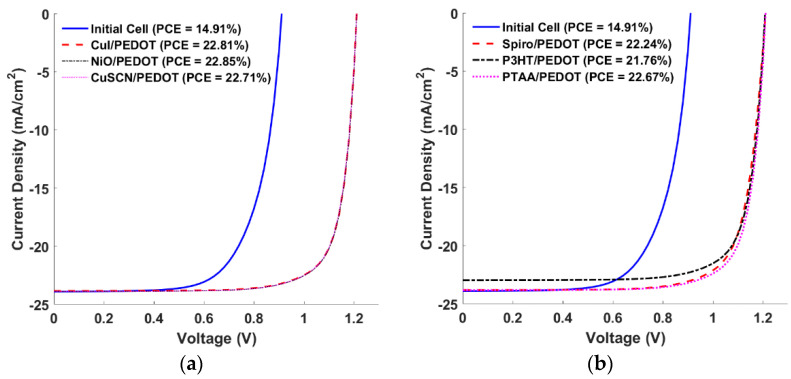
J-V curves for different double HTL materials (**a**) Inorganic/PEDOT and (**b**) Organic/PEDOT.

Now, the simulation for the different HTL materials when integrating them with PEDOT is provided, and the output J-V characteristics under illumination are shown in Figure 7. The curves of the inorganic/PEDOT are represented in Figure 7a, while those of the organic/PEDOT are illustrated in Figure 7b. The results indicate that the S-shape curve behavior has been relieved, as expected. Regarding the inorganic/PEDOT cells, the curves are almost identical, with NiO/PEDOT having a small enhancement, while for the organic/PEDOT structures, PTAA/PEDOT gives the best performance among the other cases. All the PV parameters of the studied double HTLs are summarized in Table S3 in the Supplementary Materials. It is also noticed from the results that the PCEs of the inorganic materials are higher than those of their organic partners.

**Figure 8:**
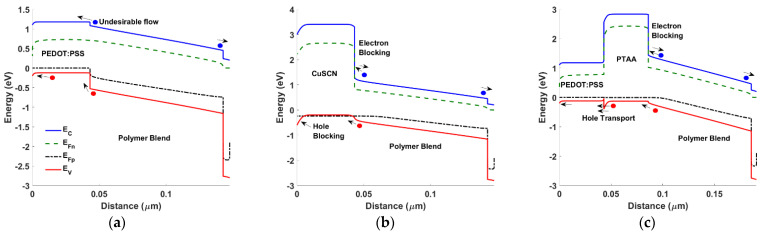
Energy band diagram at short circuit current under illumination for different HTL architectures (**a**) PEDOT, (**b**) CuSCN, and (**c**) PTAA/PEDOT.

To give a physical explanation of the trend of the results when using just one HTL and when utilizing a double HTL, the energy band diagram for three different cases is drawn, namely for single HTL PEDOT, CuSCN, and a double HTL PTAA/PEDOT, as shown in Figure 8. It can be inferred from comparing Figure 8b versus Figure 8a, that although CuSCN provides much stronger electron blocking than PEDOT, its misalignment with the ITO contact, leading to its high *φ_B_*, creates a barrier that unfavorably blocks the hole transport. On the other hand, regarding Figure 8c, the combination of two HTLs facilitates the hole transport toward the ITO contact in addition to its electron-blocking capability. Thus, the design of a double HTL achieves both a higher efficiency and a suppressed kink behavior.

### 3.3. Impact of Light Intensity

**Figure 9:**
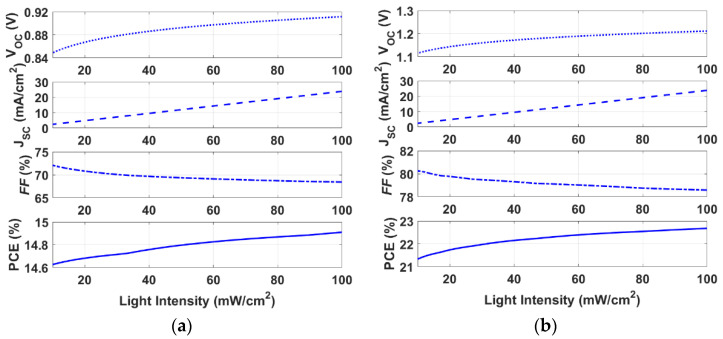
Variation of optoelectronic parameters according to the change in light intensity (**a**) initial PEDOT single HTL cell, and (**b**) PTAA/PEDOT double HTL.

Next, the polymer cell is subjected to various values of light intensity in the range of 10 to 100 mW/cm^2^ and simulated to evaluate its performance under the influence of weak light. As can be deduced from Figure 9a, the cell demonstrates a slightly lower PCE under lower light intensities in comparison to 100 mW/cm^2^ (AM1.5G) down to 10 mW/cm^2^. Although when decreasing the light intensity *V_oc_* and *J_sc_* degrade, the FF is considerably improved, and the input power is lowered, altogether resulting in a percentage decrease of only 1.9% in the efficiency of the cell (for the change in intensity from 100 to 10 mW/cm^2^). The same trend holds for the double HTL design (for PTAA/PEDOT), as illustrated in Figure 9b. In this case, the relative percentage decrease is 5.9% in the PCE. These interesting results reveal that the presented polymer cell can be said to have a favorable potential for indoor PV applications, thanks to its proper performance in low light intensity conditions. 

Finally, to offer a comparative evaluation, some published, both experimental and simulated, polymer-based SC PV parameters, including the optical band gaps, along with the presented all-polymer SC, are given in Table 5. Some of the addressed cells were measured experimentally while others were simulated. Different polymer blends are shown to have distinct bandgap energies. All the experimental and simulated cells are based on a single HTL except our proposal, which gives the highest PCE amongst the other cells. 

## 4. Conclusions

In this work, a single junction, all-polymer solar cell has been introduced and designed by employing numerical simulation performed by the SCAPS device simulator. The initial parameters of each layer in the SC were extracted from experimental work. The performance of the presented SC has been examined to understand the main roles of the barrier height between the HTL and the front ITO contact, as well as the VBO between the HTL and the absorber. The impact of different inorganic and organic HTL materials has been explored. The results showed that inorganic CuI and organic P3HT materials are the most suitable choices, as they have minor kink behavior. The highest PCE obtained was 20.87% for CuI, while it was 20.17% for P3HT. A double HTL structure has been proposed, in which the PEDOT serves as the HTL near the ITO contact to accomplish a proper barrier height and to facilitate the flow of the hole transport towards the contact. In addition, the extra HTL beside the PEDOT is in contact with the absorber. This additional HTL is designed in such a way as to achieve a suitable VBO for the hole flow. The simulation results revealed that this suppressed the kink behavior. The systems based on NiO/PEDOT and PTAA/PEDOT give the best performance among the other cases, where PCEs of 22.85% and 22.67% were achieved, respectively. Moreover, the response of the SC was investigated when decreasing the light intensity, and the results show a promising performance, signifying that the designed all-polymer SC could be efficiently developed for indoor photovoltaic applications. The presented simulation study can open potential paths to design all-polymer SCs that have low costs and high efficiencies.

## Figures and Tables

**Table 1:** Main input parameters of the various layers of the polymer-based solar cell [24,33,42].

Parameters	PEDOT:PSS	Polymer Blend	PFN-Br
*t* (nm)	40	100	5
LUMO (eV)	3.60	3.69	3.90
HOMO (eV)	4.90	5.30	6.90
*ε_r_*	3.5	3.5	3.5
*µ_n_* (cm^2^/V∙s)	8.0 × 10^−4^	5.13 × 10^−4^	1.0 × 10^−4^
*µ_p_* (cm^2^/V∙s)	8.0 × 10^−4^	2.53 × 10^−4^	1.0 × 10^−6^
*N_c_* (cm^−3^)	1 × 10^21^	1 × 10^21^	1 × 10^21^
*Nv* (cm^−3^)	1 × 10^21^	1 × 10^21^	1 × 10^21^
*N_D_* (cm^−3^)	-	-	-
*N_A_* (cm^−3^)	1 × 10^19^	-	-

**Table 2:** Main factors of the top and back metal contacts [41].

	Material	Work Function (eV)	*S_n_* (cm/s)	*S_p_* (cm/s)
Front Metal	ITO	4.7	10^7^	10^5^
Back Metal	Ag	4.1	10^5^	10^7^

**Table 3:** Solar cell key metrics extracted from illuminated *J-V* characteristics for both experimental and calculated data.

	*V_OC_* [V]	*J_SC_* [mA/cm^2^]	FF [%]	PCE [%]
Measurements [24]	0.912	23.90	68.50	14.90
SCAPS Simulation	0.912	23.91	68.45	14.91

**Table 4:** Main input physical parameters of various HTL materials.

	CuI [49,50]	NiO [49,50]	CuSCN [49,50]	Spiro-OMeTAD [50]	P3HT [50,52]	PTAA [50,51]
*E_g_* (eV)	3.10	3.80	3.60	2.9	2.0	2.96
χ (eV)	2.10	1.46	1.70	2.2	3.20	2.30
VBO (eV)	−0.1	−0.04	0	−0.2	−0.1	−0.04
*φ_B_* (eV)	0.498	0.555	0.642	0.398	0.379	0.439
*ε_r_*	6.5	10.7	10	3.0	3.0	9.0
*µ_e_* (cm^2^/V∙s)	100	12	100	1 × 10^−4^	1 × 10^−4^	1 × 10^−4^
*µ_h_* (cm^2^/V∙s)	43.9	2.8	25	2 × 10^−4^	1 × 10^−3^	4 × 10^−3^
*N_c_* (cm^−3^)	2.8 × 10^19^	2.8 × 10^19^	2.2 × 10^19^	2.8 × 10^19^	1 × 10^21^	1 × 10^21^
*N_v_* (cm^−3^)	1.0 × 10^19^	1.0 × 10^19^	1.8 × 10^18^	1.0 × 10^19^	1 × 10^21^	1 × 10^21^

**Table 5:** State-of-the-art comparison between some experimental and simulated polymer-based SCs, showing the main PV performance parameters.

Active Blend	HTL	ETL	*V_OC_* (V)	*J_SC_* (mA/cm^2^)	FF (%)	PCE (%)	*E_g_* (eV)	Notes	REF
PM6:Y6	PEDOT	PFN-Br	0.842	26.05	72.03	15.79	1.38	Exp.	[14]
PBDB-T:PZT-γ	PEDOT	PFN-Br	0.896	24.70	71.30	15.80	1.51	Exp.	[23]
PBDB-T:PZT-C1	PEDOT	PFN	0.912	23.90	68.50	14.90	1.61	Exp.	[24]
PBDB-T:PN-Se	PEDOT	PDINN	0.907	24.82	71.80	16.16	1.51	Exp.	[57]
PM6:PY-IT	PEDOT	PDINN	0.950	23.95	72.05	16.41	1.75	Exp.	[58]
PY-IT/BNT: PM6	PEDOT	ZrAcAc	0.96	22.7	0.74	16.09	1.45	Exp.	[59]
PY2F-T:PYT:PM6	PEDOT	PDIN	0.90	25.2	76.0	17.2	1.343	Exp.	[60]
D18:N3	PEDOT	PDIN	0.862	27.44	78.5	18.56	1.63	Exp.	[61]
PTB7:PC_70_BM	PEDOT	PFN-Br	0.731	16.43	68.05	8.18	0.90	Sim.	[33]
PTB7:PC_70_BM	PEDOT	Zn(O,S)	0.855	28.37	70.69	17.15	1.10	Sim.	[34]
PBDB-T:PZT	CuI	PFN	1.209	23.87	72.28	20.87	1.61	Single HTL	This work
PBDB-T:PZT	PEDOT/NiO	PFN	1.212	23.88	78.95	22.85	1.61	Double HTL	This work

Sim. = simulation and Exp. = experiment.

## Data Availability

No new data were created or analyzed in this study. Data sharing does not apply to this article.

## Supplementary Materials

The following supporting information can be downloaded at: https://www.mdpi.com/article/10.3390/polym15040869/s1, Figure S1: SCAPS simulator flowchart showing the basic semiconductor equations and the different required input parameters; Table S1: Definitions of SCAPS input and output parameters; Table S2: Main PV parameters for various single HTL materials; Table S3: Main PV parameters for various double HTL materials (integrated with PEDOT).
