# Supplementary material for: Device Modeling of Efficient PBDB-T:PZT-Based All-Polymer Solar Cell: Role of Band Alignment
Source: Polymers (Basel). 2023 Feb 9;15(4):869. doi: 10.3390/polym15040869 (PMC9967467; doi:10.3390/polym15040869)
Supplement: Supplementary file 1 [file polymers-15-00869-s001.zip › polymers-2179380-supplementary.pdf]

polymers

MDPI

Supplementary Materials

Device Modeling of Efficient PBDB-T:PZT-Based All-Polymer Solar Cell: Role of Band Alignment

Marwa S. Salem [1,2], Ahmed Shaker [3] and Mostafa Mohamed Salah [4,*]

1 Department of Computer Engineering, College of Computer Science and Engineering, University of Ha'il, Ha'il 55211, Saudi Arabia
2 Department of Electrical Communication and Electronics Systems Engineering, Faculty of Engineering, Modern Science and Arts University, Cairo 12556, Egypt
3 Faculty of Engineering, Ain Shams University, Cairo 11535, Egypt
4 Electrical Engineering Department, Future University in Egypt, Cairo 11835, Egypt
* Correspondence: mostafa.abdulkhalek@fue.edu.eg

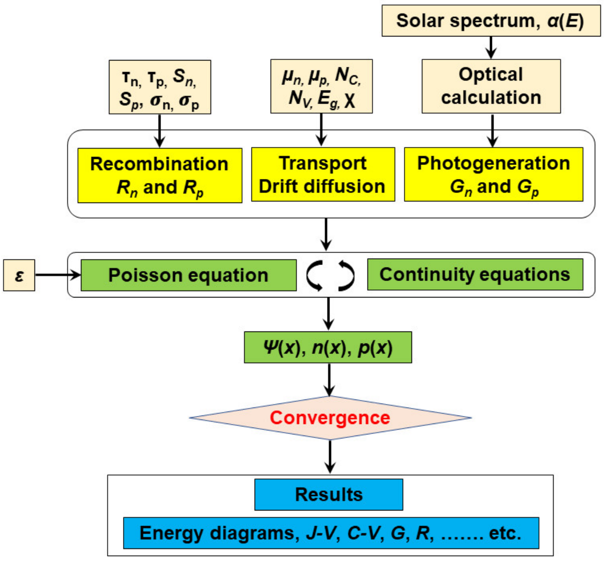

$$J_n = -\frac{n\mu_n}{q}\frac{\partial E_{Fn}}{\partial x}$$

$$J_p = +\frac{p\mu_p}{q}\frac{\partial E_{Fp}}{\partial x}$$

$$\frac{\partial^2\psi}{\partial x^2} = -\frac{q}{\varepsilon_r\varepsilon_0}(p - n + N_D^+ - N_A^- \pm N_t)$$

$$\left(\frac{1}{q}\right)\frac{\partial J_n}{\partial x} - R_n(n,p) + G = 0$$

$$-\left(\frac{1}{q}\right)\frac{\partial J_p}{\partial x} - R_p(n,p) + G = 0$$

Figure S1. SCAPS simulator flowchart showing the basic semiconductor equations and the different required input parameters.

Table S1. Definitions of SCAPS input and output parameters.

Parameter.	Description	Unit	Parameter	Description	Unit
t	Thickness	nm	q	electrical charge	C
E_g	Energy gap	eV	ψ	Electrostatic potential	V
χ	Electron affinity	eV	n	Electron concentration	cm^{-3}
ε_r	Relative permittivity		p	Hole	cm^{-3}
μ_n	Electron mobility	cm^2/V·s	J_n	Electron current density	mA/cm^2
μ_p	Hole mobility	cm^2/V·s	J_p	Hole current density	mA/cm^2
N_c	Conduction band effective DOS	cm^{-3}	N_t	Defect density	cm^{-3}
N_v	Valence band effective DOS	cm^{-3}	G	Generation rate	cm^{-3} s^{-1}
N_D^+	Ionized donor density	cm^{-3}	R	Recombination rate	cm^{-3} s^{-1}
N_A^-	Ionized acceptor density	cm^{-3}	E_{Fn}, E_{Fp}	Quasi-Fermi energy levels	eV

Table S2. Main PV parameters for various single HTL materials.

Performance Parameters	PEDOT	CuI	NiO	CuSCN	Spiro	P3HT	PTAA
V_{OC} [V]	0.912	1.209	1.211	1.183	1.193	1.191	1.204
J_{SC} [mA/cm^2]	23.91	23.875	23.906	23.901	23.842	22.985	23.851
FF [%]	68.45	72.28	67.75	62.98	69.13	73.69	70.64
PCE [%]	14.91	20.87	19.61	17.81	19.67	20.17	20.29

Table S3. Main PV parameters for various double HTL materials (integrated with PEDOT).

Performance Parameters	CuI	NiO	CuSCN	Spiro	P3HT	PTAA
V_{OC} [V]	1.212	1.212	1.212	1.211	1.209	1.211
J_{SC} [mA/cm^2]	23.848	23.880	23.877	23.815	22.967	23.824
FF [%]	78.93	78.95	78.47	77.13	78.36	78.57
PCE [%]	22.81	22.85	22.71	22.24	21.76	22.67

Materials' Abbreviations.

Abbr.	Full Name
PBDB-T	Poly[(2,6-(4,8-bis(5-(2-ethylhexyl)thiophen-2-yl)-benzo[1,2-b:4,5-b']dithiophene))-alt-(5,5-(1',3'-di-2-thienyl-5',7'-bis(2-ethylhexyl)benzo[1',2'-c:4',5'-c']dithiophene-4,8-dione)]
PZT	lead zirconate titanate (Pb[Zr(x)Ti(1-x)]O3)
ITO	Indium tin oxide
PEDOT:PSS	poly(3,4-ethylenedioxythiophene) polystyrene sulfonate
PFN-Br	Poly(9,9-bis(3'-(N,N-dimethyl)-N-ethylammoinium-propyl-2,7-fluorene)-alt-2,7-(9,9-di-octylfluorene))dibromide
CuI	Copper Iodide
P3HT	Regioregular poly(3-hexylthiophene)
PCBM	fullerene derivative [6,6]-phenyl-C_{61}-butyric acid methyl ester
ZnOS	Zinc oxysulphide
Ag	Silver
NiO	Nickel oxide
CuSCN	Cuprous thiocyanate Copper(I) thiocyanate
Spiro-OMeTAD	[2,2′,7,7′-tetrakis(N,N-di-p-methoxyphenyl-amine)9,9′-spirobifluorene]
PTAA	poly(triaryl amine) semiconductor
PM6	Poly[(2,6-(4,8-bis(5-(2-ethylhexyl-3-fluoro)thiophen-2-yl)-benzo[1,2-b:4,5-b']dithiophene))-alt-(5,5-(1',3'-di-2-thienyl-5',7'-bis(2-ethylhexyl)benzo[1',2'-c:4',5'-c']dithiophene-4,8-dione)]
Y6	thienothienopyrrolo-thienothienoindole (TTP-TTI) core base and 2-(5,6-difluoro-3-oxo-2,3-dihydro-1H-inden-1-ylidene)malononitrile (2FIC)
PN-Se	Polyphosphazenes-selenium
PDINN	N,N'-Bis{3-[3-(Dimethylamino)propylamino]propyl}perylene-3,4,9,10-tetracarboxylic diimide
PY	Pyridine
PTB7	Poly [[4,8-bis[(2-ethylhexyl)oxy]benzo[1,2-b:4,5-b']dithiophene-2,6-diyl][3-fluoro-2-[(2-ethylhexyl)carbonyl]thieno[3,4-b]thiophenediyl]]
ZrAcAc	zirconium acetylacetonate
D18	Poly[(2,6-(4,8-bis(5-(2-ethylhexyl-3-fluoro)thiophen-2-yl)-benzo[1,2-b:4,5-b']dithiophene))-alt-5,5'-(5,8-bis(4-(2-butyloctyl)thiophen-2-yl)dithieno[3',2':3,4;2'',3'':5,6]benzo[1,2-c][1,2,5]thiadiazole)]
N3	azide Azide ion Hydrazoate Azide anion
